# Supplementary material for: Wood hemicelluloses exert distinct biomechanical contributions to cellulose fibrillar networks
Source: Nat Commun. 2020 Sep 17;11:4692. doi: 10.1038/s41467-020-18390-z (PMC7499266; doi:10.1038/s41467-020-18390-z)
Supplement: Supplementary file 1 — Supplementary Information [file 41467_2020_18390_MOESM1_ESM.pdf]

Supplementary Information

Wood Hemicelluloses Exert Distinct Biomechanical Contributions to Cellulose Fibrillar Networks

Jennie Berglund[1], Deirdre Mikkelsen[2,3], Bernadine M. Flanagan[2], Sushil Dhital[2,4], Stefan Gaunitz[5], Gunnar Henriksson[1], Mikael E. Lindström[1], Gleb E. Yakubov[6,7,*], Michael J. Gidley[2,*], and Francisco Vilaplana[1,5,*]

[1]Wallenberg Wood Science Centre, Department of Fibre and Polymer Technology, School of Engineering Sciences in Chemistry, Biotechnology and Health, KTH Royal Institute of Technology, Stockholm, Sweden

[2]ARC Centre of Excellence in Plant Cell Walls, Centre for Nutrition and Food Sciences, Queensland Alliance for Agriculture and Food Innovation, The University of Queensland, St. Lucia, Brisbane, QLD 4072, Australia

[3]School of Agriculture and Food Sciences, The University of Queensland, St. Lucia, Brisbane, QLD 4072, Australia

[4]Department of Chemical Engineering, Monash University, Clayton, VIC 3800, Australia

[5]Division of Glycoscience, Department of Chemistry, School of Engineering Sciences in Chemistry, Biotechnology and Health, AlbaNova University Centre, KTH Royal Institute of Technology, Stockholm, Sweden

[6]ARC Centre of Excellence in Plant Cell Walls, School of Chemical Engineering, The University of Queensland, St. Lucia, Brisbane, QLD 4072, Australia.

[7]School of Biosciences, Faculty of Sciences, University of Nottingham, Nottingham, United Kingdom

*Corresponding authors: Francisco Vilaplana (franvila@kth.se), Michael J. Gidley (m.gidley@uq.edu.au), Gleb Yakubov (gleb.yakubov@nottingham.ac.uk)

Supplementary Methods

Linear biphasic model: analysis of compression-stress relaxation profiles

The composites are assumed to be transversely isotropic in the x,y-plane. This approximation was found to be adequate for describing bacterial cellulose hydrogels given that they are roughly produced in a layer-by-layer fashion.[1,2] In this work we have utilized two models of the linear poroelastic theory: confined and unconfined compression developed by Mow et al.[3] and Cohen et al.[4], respectively. In our setup, the sides of the hydrogels are not bound by a container wall, and hence water is free to move in a similar manner to unconfined compression setup. The use of sandpaper, however, restricts the lateral expansion of hydrogels, making the balance of stresses within the sold network similar to that described by the confined compression model. In addition, the roughness of the top and bottom plates can facilitate water drainage, making the hydrogels respond in a similar way to the confined compression experiment,[3] where the top plate made of porous material allows water drainage out of the hydrogel. The results of unconfined compression/relaxation modelling were less successful compared to the results obtained using the confined compression/relaxation model. To further refine the confined compression model we have introduced an *ad hoc* modification to account for the fact that drainage of the fluid occurs through the sides of the hydrogels as well as through the top surfaces in contact with sandpaper.[5,6] According to this model, the normal stress $\sigma_n(t)$ resulting from a ramp displacement in the z direction at constant strain rate $\dot{\varepsilon}_0$ during t_0 seconds, followed by a relaxation stage at constant strain is given by

$$\sigma_n(t) = H_A \dot{\varepsilon}_0 t + \frac{2\eta \dot{\varepsilon}_0 h_0^2}{k\pi^2}\left(\frac{\pi^2}{6} - \sum_{j=1}^{\infty} \frac{e^{-\frac{j^2 t}{t_p}}}{j^2}\right) \quad \text{Supplementary Equation 1}$$

for $0 < t < t_0$

And

$$\sigma_n(t) = H_A \dot{\varepsilon}_0 t_0 - \frac{2\eta \dot{\varepsilon}_0 h_0^2}{k\pi^2}\left(\sum_{j=1}^{\infty} \frac{e^{-\frac{j^2 t}{t_p}} - e^{-\frac{j^2 (t-t_0)}{t_p}}}{j^2}\right) \quad \text{Supplementary Equation 2}$$

for $t > t_0$

Where

$$t_p = \frac{\eta h_0^2}{E_z k \pi^2} \quad \text{Supplementary Equation 3}$$

is poroelastic time, t is time, k is the permeability, η is fluid viscosity, h_0 is sample thickness, H_A and E_z are the aggregate modulus and out-of-plant modulus, respectively. The aggregate modulus is the function of the lateral (in-plane) modulus (E_L), where σ_{zz} and ε_{zz} are stress and strain in the out-of-plane direction (i.e. direction of the applied force), C_{33} is the component of the elastic stiffness tensor, ν_L and $\nu_{Lz} = {}^{E_L}/_{E_z}\, \nu_{zL}$ are the in-plane and out-of-plane Poisson's ratios, respectively.

$$H_A = \frac{\sigma_{zz}}{\varepsilon_{zz}} = C_{33} = E_L\left(\frac{1-\nu_L}{(1-\nu_L)\,{}^{E_L}/_{E_z} - 2\nu_{Lz}^2}\right) \quad \text{Supplementary Equation 4}$$

The representative fits using the model described in Supplementary Equation 1-4 are shown in Supplementary Figure 9.

Analysis and discussion regarding anisotropy of the BC-H hydrogels

Zener ratio (*a*)

One of the possible ways of estimating the degree of anisotropy is through evaluating the effective anisotropy ratio *a* (Zener ratio), which within limits of linear elastic approximation can be defined as:

$$a_1 = \frac{2(1+v)G'}{E_{relax}} \qquad \text{Supplementary Equation 5}$$

Here we assume that $G' >> G''$ and, hence, $G \approx G'$ as well as that E can be approximated by E_{relax}. The $a = 1$ corresponds to the isotropic material, while for anisotropic materials $a > 1$. In order to estimate *a* using Supplementary Equation 5, the Poisson's ratio (v) is assumed to be 0.3. The resulting values of *a* are found to be as high as 7 – 27 depending on compression ratio and BC-H material. These estimates indicate strong anisotropy.

Modelling was applied to evaluate the poroelastic behavior of hydrogels and the aggregate modulus (H_A) was determined. Using the values of H_A, we can estimate anisotropy by evaluating effective anisotropy ratio (a_2) defined as:

$$a_2 = \frac{2(1+v)G'}{H_A} \qquad \text{Supplementary Equation 6}$$

In Supplementary Equation 6 the aggregate modulus (H_A) is compared with G' values; both quantities reflect the resistance of the material to the in-plane components of the deformation tensor. In addition, another anisotropy ratio (a_3) was introduced to estimate the impact of compression on changes in measured G' and E_{relax} ($\approx E_z$) (Supplementary Equation 7). The differential quantity $G' - G'_0$ comprises the values of G'_0 recorded at $F_n = 0$, where the top plate of the measuring geometry was still in contact with the pellicle, as well as values of G' recorded at fixed values of applied load, for which the corresponding out-of-plane moduli (E_{relax}) were evaluated. Here, the quantity $G' - G'_0$ is used as a proxy value for estimating contribution of out-of-plane components of the deformation vector on the material resistance to the compression.

$$a_3 = \frac{2(1+v)(G'-G'_0)}{E_{relax}} \qquad \text{Supplementary Equation 7}$$

All anisotropy ratios (a_1, a_2, and a_3) calculated at a CR around 0.1 are presented in Supplementary Table 3. The values of a_2 are found to be close to 1, indicating that H_A and G' show prominent correspondence and may describe the mechanical response of cellulose fibers predominantly oriented in the horizontal direction of the BC and BC-H materials. The a_3 values are found to be markedly lower compared to a_1 values (19-54 % reduction), which suggests that increase in G' upon compression is partially accounted for by the response of the deformed fibers aligned in the out-of-plane direction of the hydrogel.

Supplementary Figures

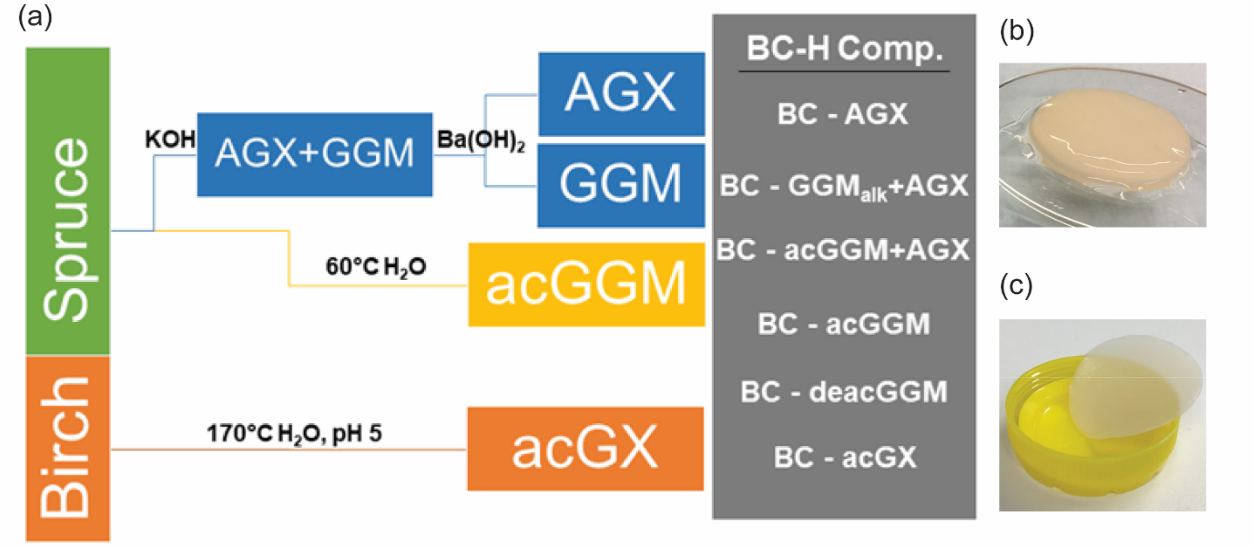

Supplementary Figure 1. Extraction of wood hemicelluloses and list of fabricated BC-H composites. (a) Schematic overview of hemicellulose extraction processes. (b & c) Photos of a BC pellicle after harvest (b) and after compression (c), representing the typical macro-structures observed after these processes, for all the harvested materials fabricated.

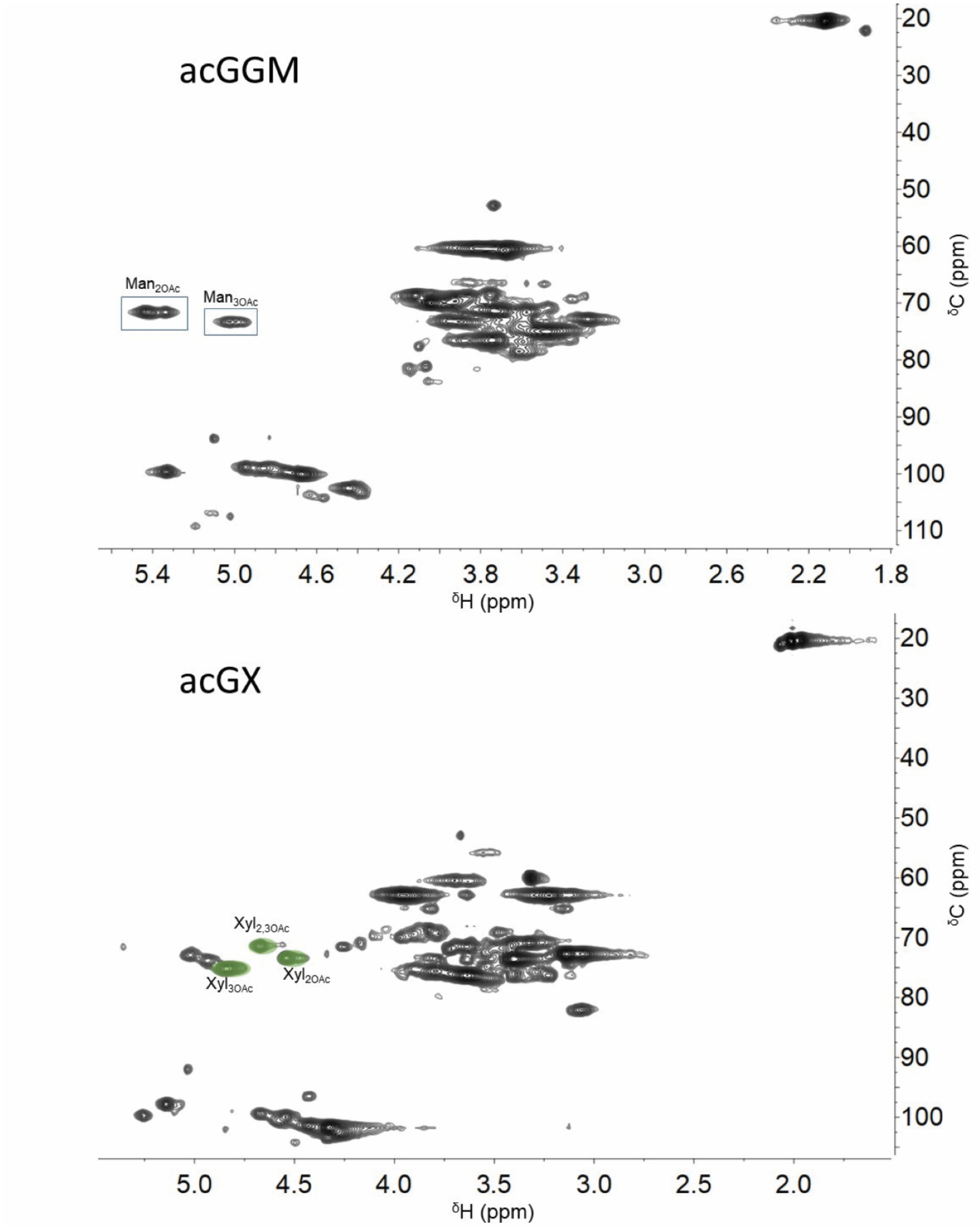

Supplementary Figure 2. HSQC 2D NMR of the acetylated hemicelluloses acGGM and acGX. Man_{2OAc} = *O*-acetylation at C2 of mannose, Man_{3OAc} = *O*-acetylation at C3 of mannose, Xyl_{2OAc} = *O*-acetylation at C2 of xylose, Xyl_{3OAc} = *O*-acetylation at C3 of xylose, Xyl_{23OAc} = *O*-acetylation at C2 and C3 in the same xylose unit.

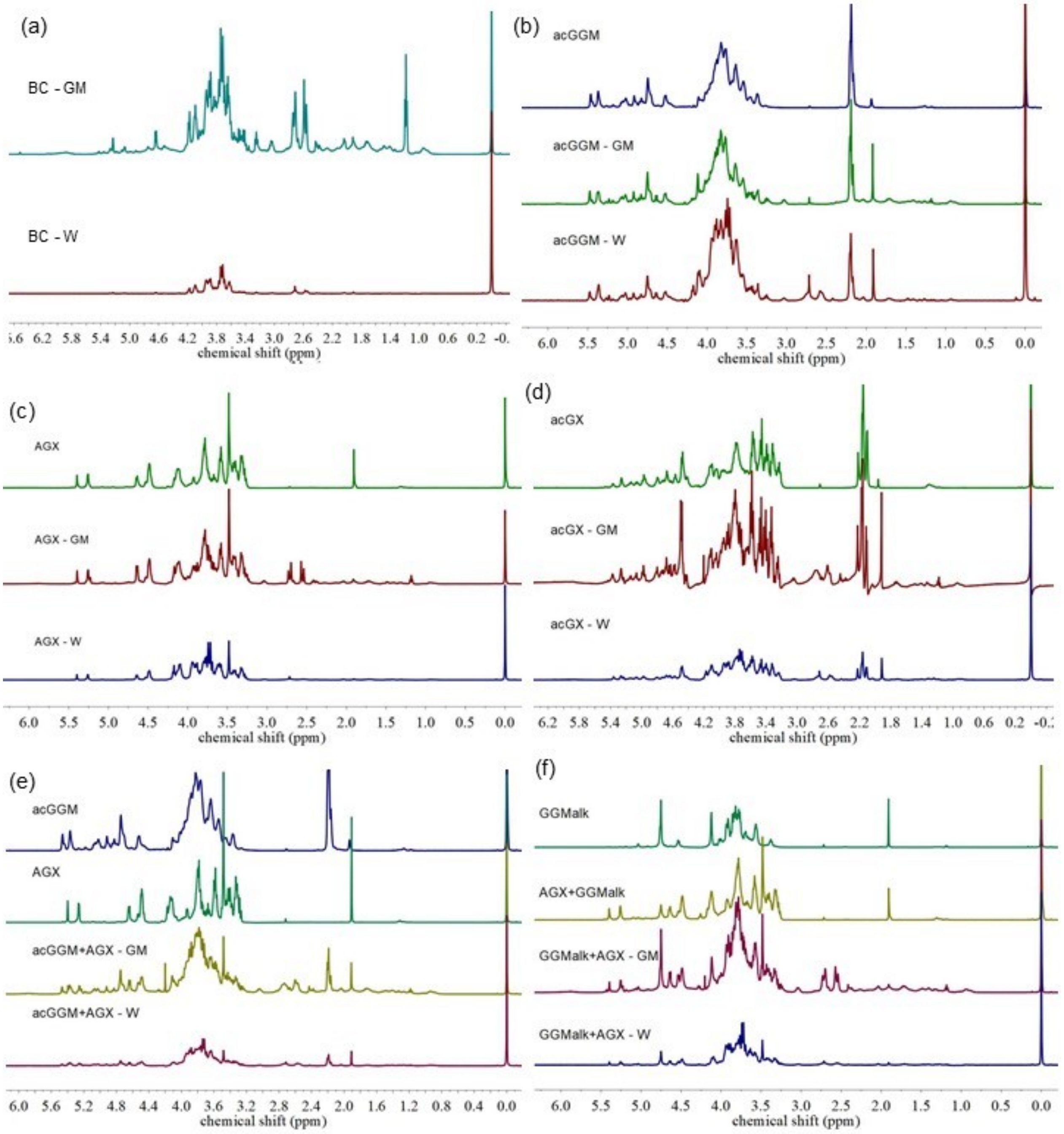

Supplementary Figure 3. Solution ^{1}H NMR of wood hemicelluloses. We compare the extracted wood hemicelluloses and the hemicelluloses left in the wash water (W) and the growth medium (GM) after harvest showing that the sugar ratios remain unaltered. (a) bacterial cellulose (BC-GM and BC-W); (b) spruce glucomannan (acGGM, acGGM-GW and acGGM-W); (c) spruce xylan (AGX, AGX-GW, AGX-W); (d) birch xylan (acGX, acGX-GM, acGX-W); (e) spruce hemicellulose mixture (acGGM, AGX, acGGM+AGX-GM, acGGM+AGX-W); (f) alkaline spruce hemicelluloses (GGMalk, AGX+GGMalk, GGMalk+AGX-GM, GGMalk+AGX-W).

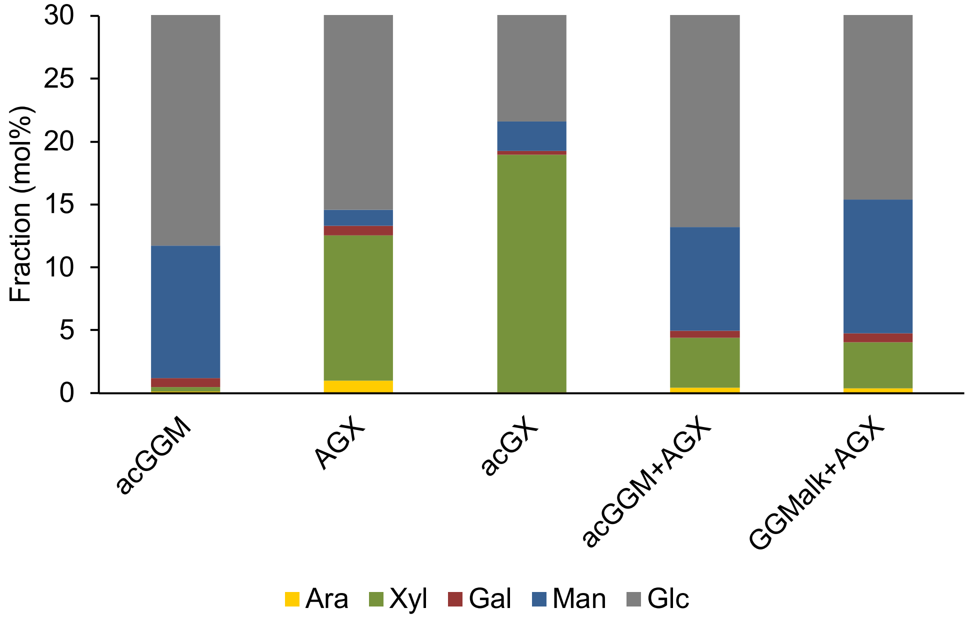

Supplementary Figure 4. Monosaccharide composition (mol%) of compressed tensile tested BC-H composites. Samples were analyzed in duplicates and the standard deviation was 0.00-0.84.

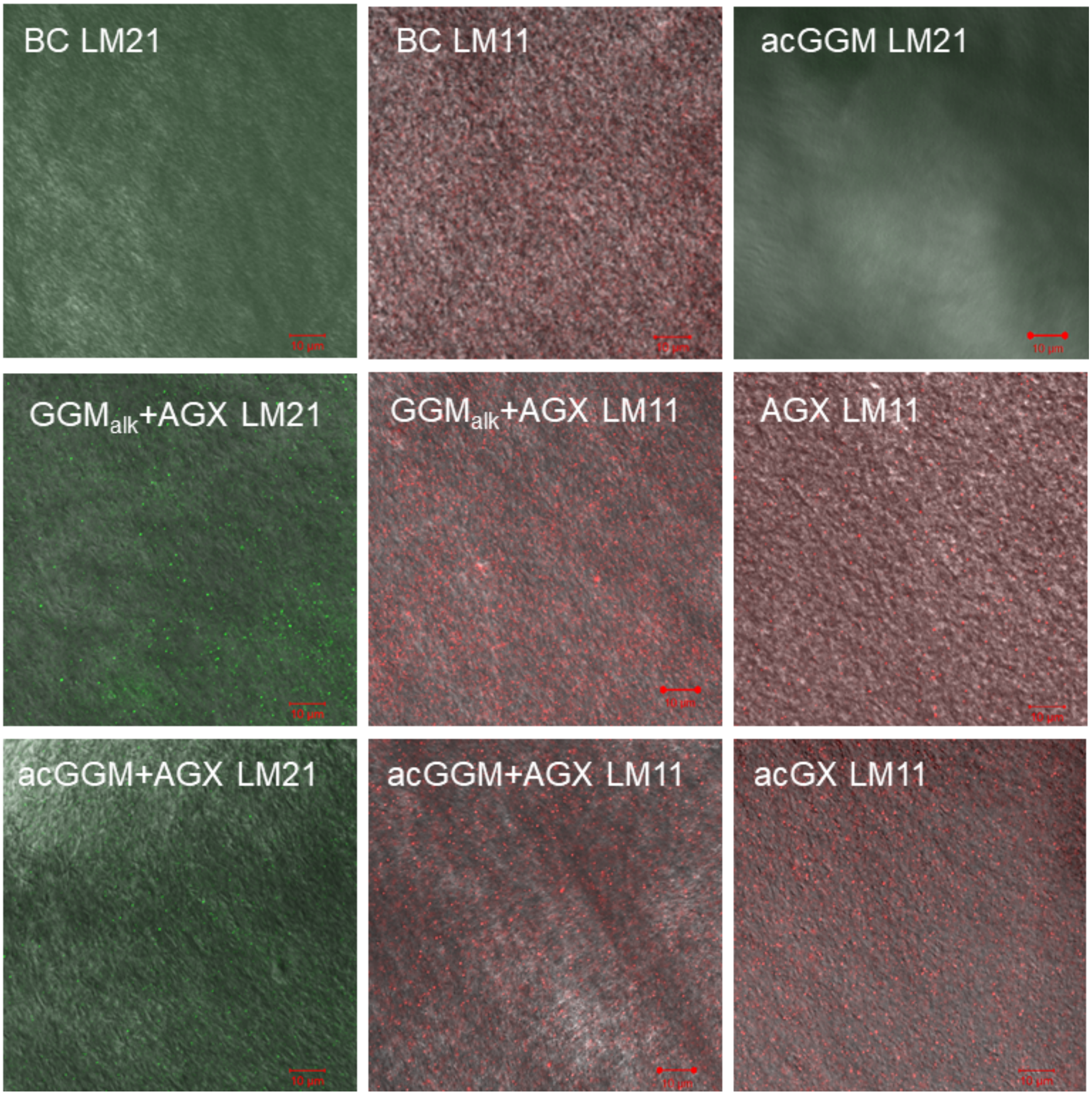

Supplementary Figure 5. Antibody labelled materials and images from the confocal microscope. Xylan is labelled by LM11 (showed in red). Glucomannans are labelled by LM21 (shown in green). The length of the scale bar corresponds to 10 μm.

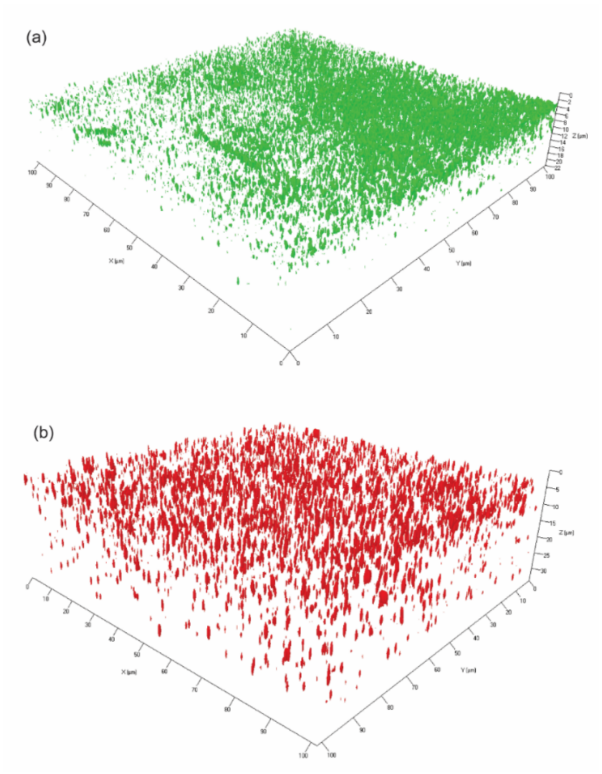

Supplementary Figure 6. 3D-images of antibody labelled BC-H. (a) LM21 of BC-GGM_{alk}+AGX (b) LM11 of BC-AGX.

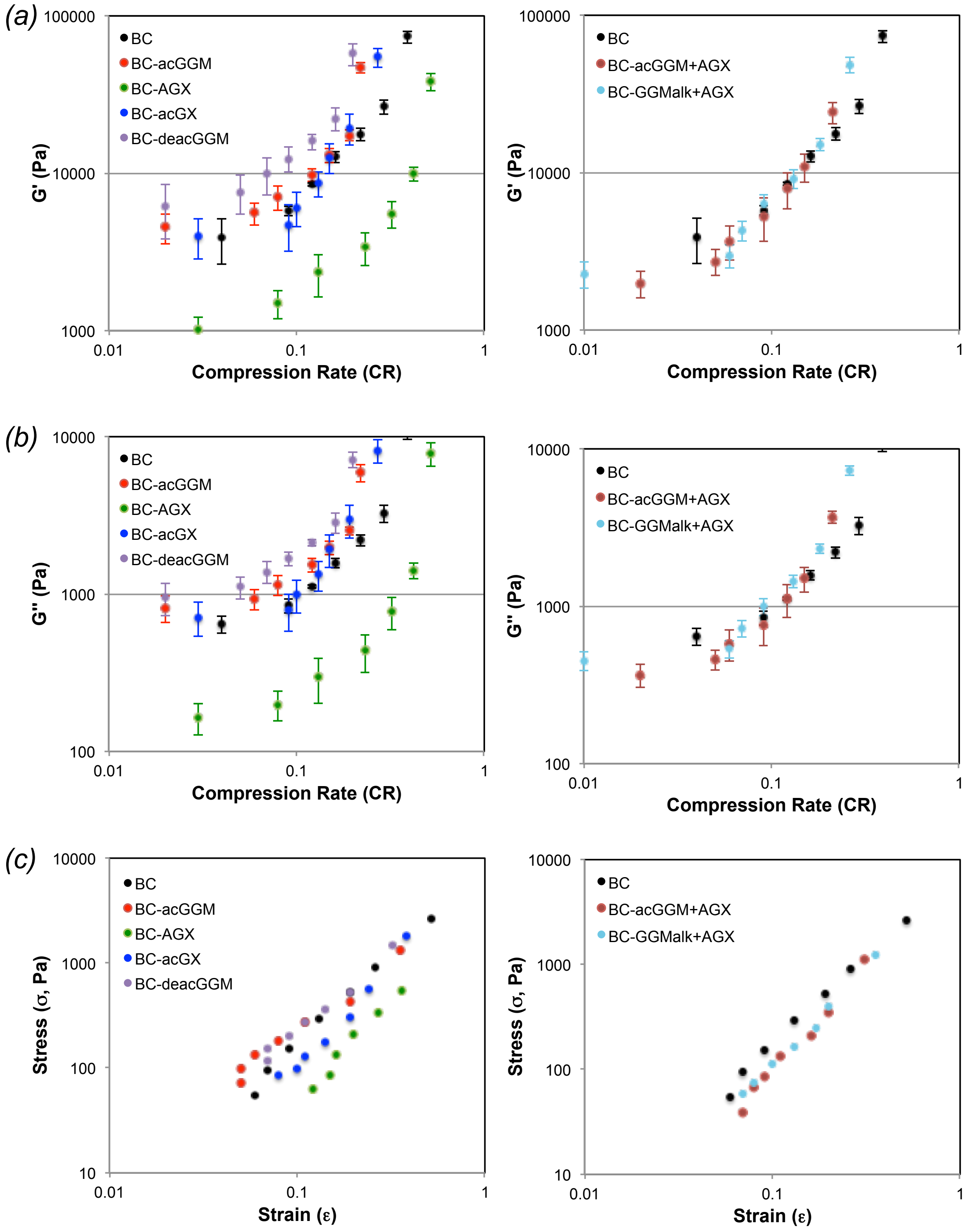

Supplementary Figure 7. Compression - relaxation mechanical analysis for the BC-H hydrogels. (a) Storage modulus (G') as a function of compression rate. (b) Loss modulus (G'') as a function of compression rate. (c) Stress – strain curves. The error bars correspond with the standard deviation (n=3). Source data are provided as a Source Data file.

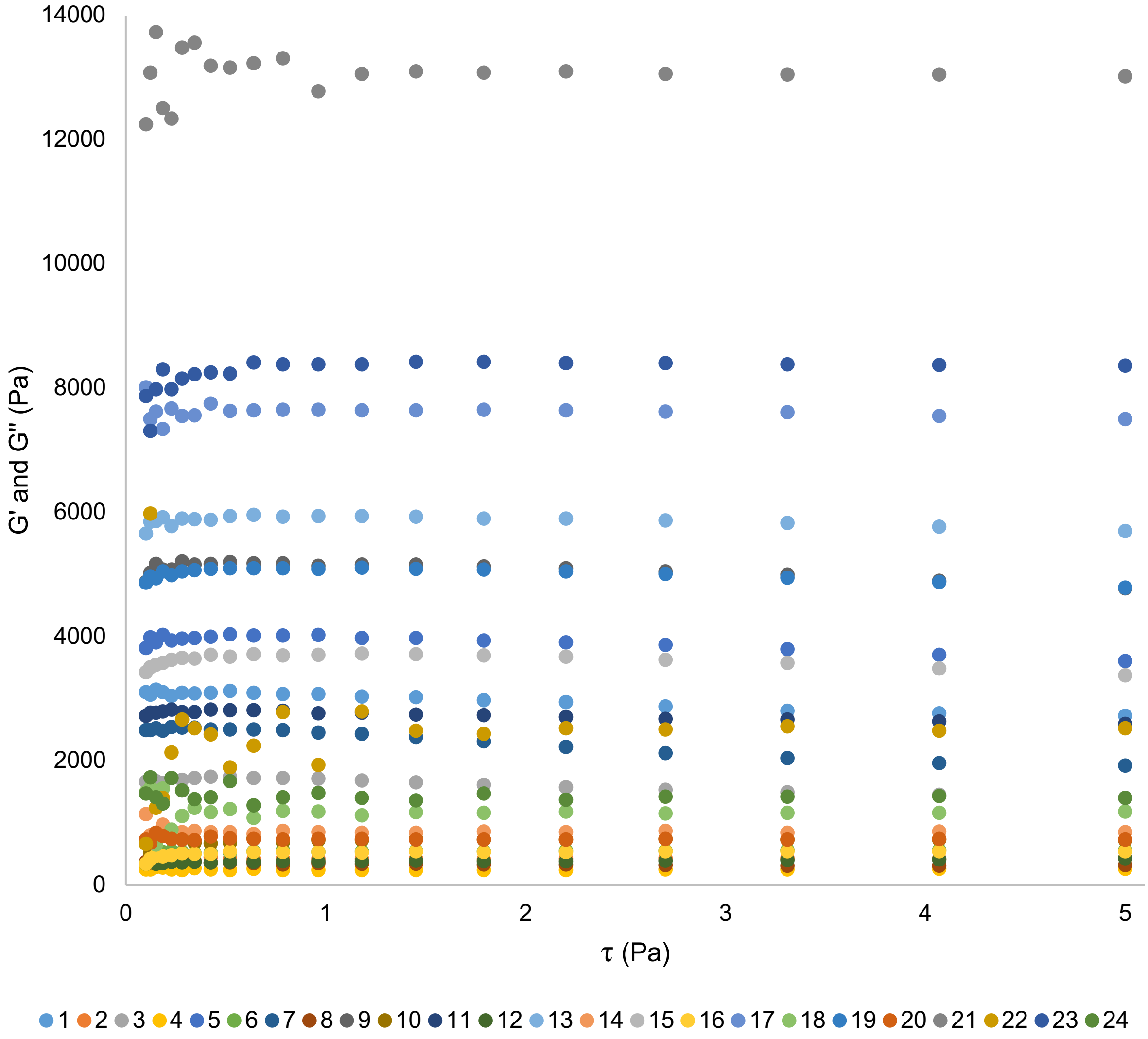

Supplementary Figure 8. Stress sweep data determining the linear viscoelastic region for BC. Each color represents values from the same sweep between 0.1 and 5 Pa at different compression ratios. The region of 2-4 Pa was selected due to linear behavior in all cases. τ = shear stress.

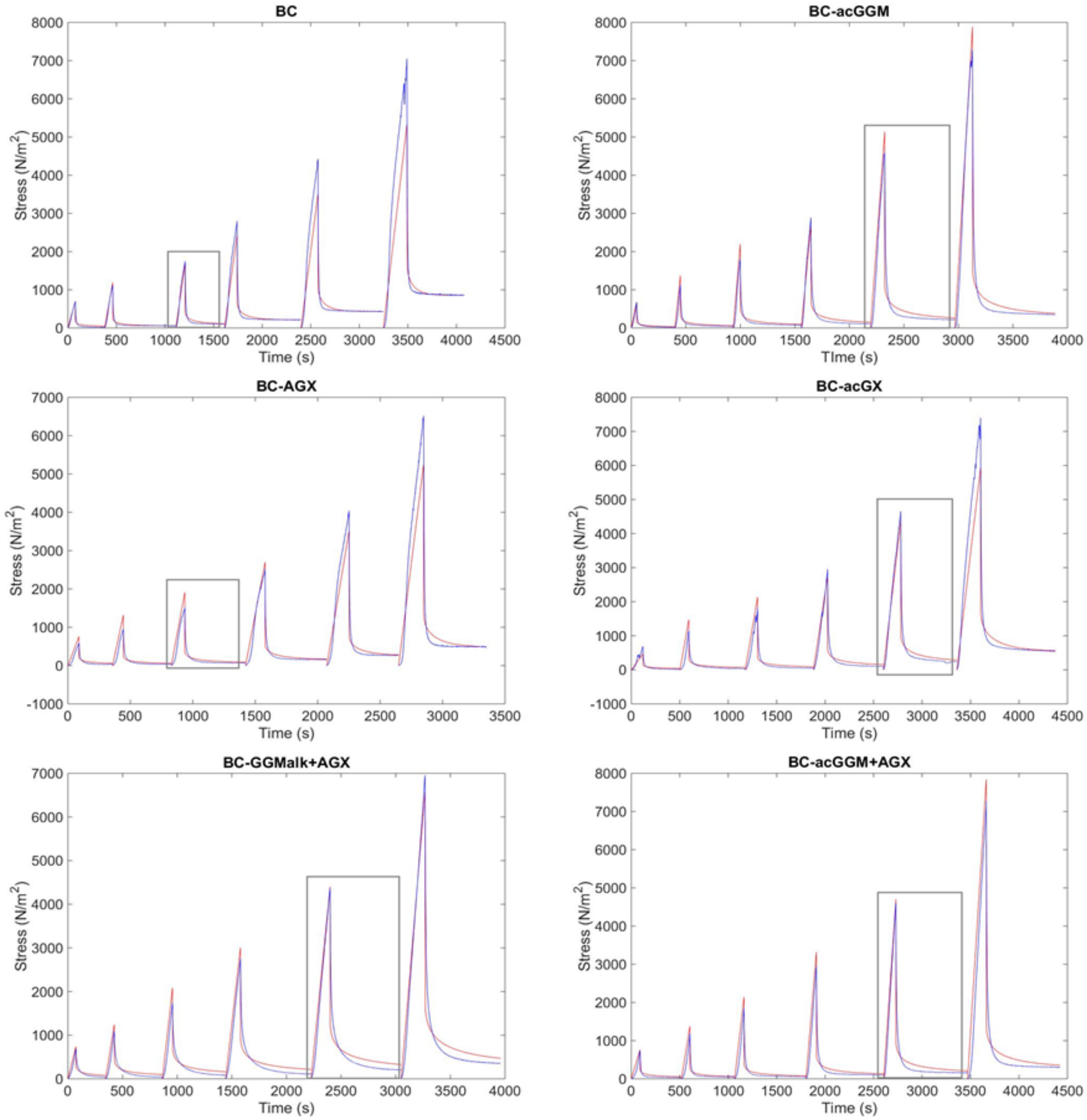

Supplementary Figure 9. Modelled fit to compression raw data. Raw data in blue and model fit in red. The compression cycles correspond to 1, 1.6, 2.5, 4, 6.3 and 10 N, and the one highlighted in the gray box is at a CR ≈ 0.1.

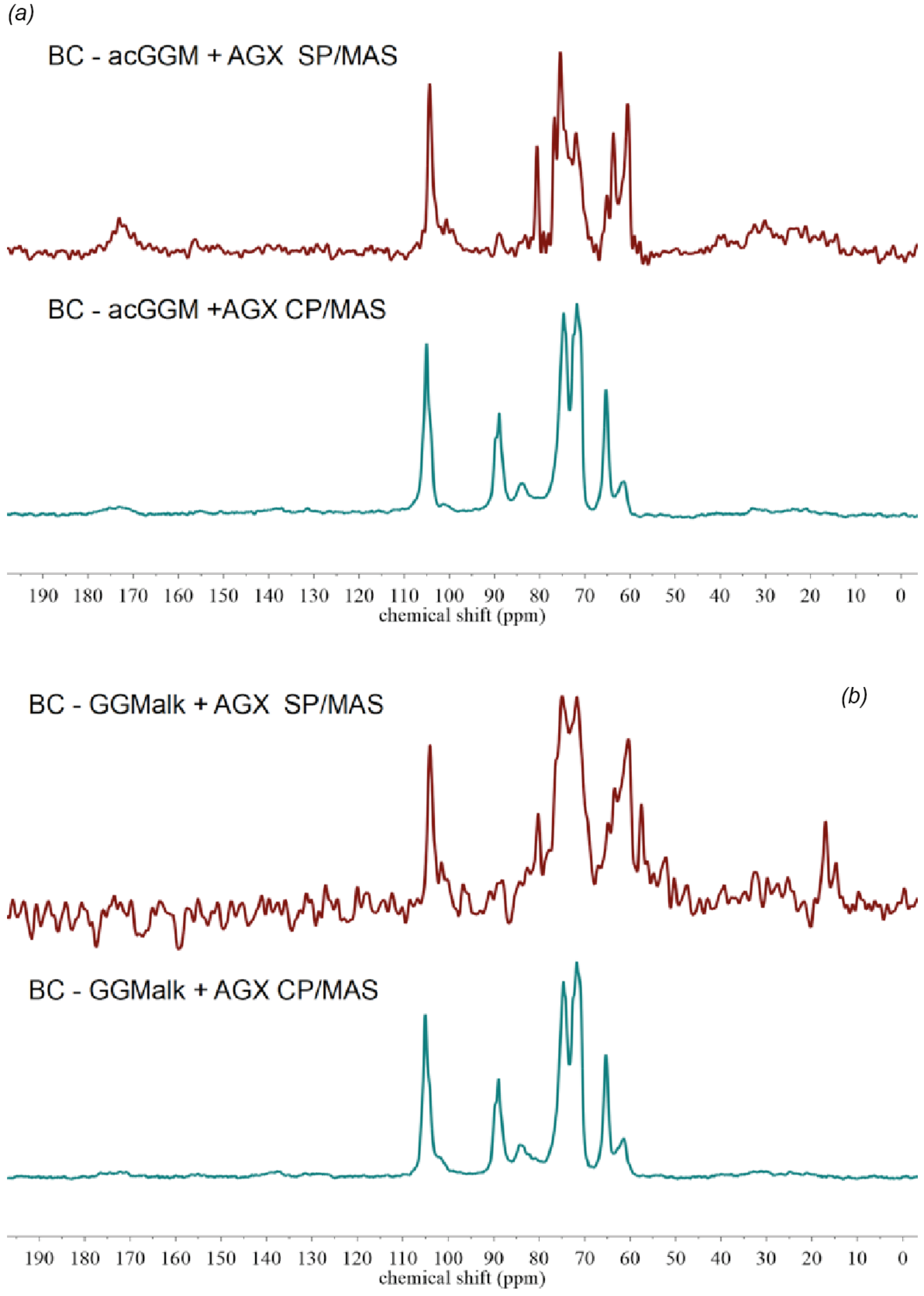

Supplementary Figure 10. Comparison of the solid-state ^{13}C CP/MAS and SP/MAS NMR spectra for the ternary BC-H hydrogels: (a) BC-acGGM+AGX; (b) BC-GGMalk+AGX.

Supplementary Tables

Supplementary Table 1. Characterization of extracted wood hemicelluloses. Characterizations of extracted materials including DS_{ac} (standard deviation: ±0.00-0.01), acetyl distribution on C2 and C3 (estimated from HSQC 2D NMR, *around 16 % of acetylated Xyl units are acetylated at both C2 and C3), weight average molecular weight (M_w) determined by SEC (0.1 M $NaNO_3$ + 5 mM NaN_3) with standard calibration (StC) and light scattering calibration (LsC), and monosaccharide composition (standard deviation: ±0.0-2.6).

**Hemicellulose sample**	**acGGM**	**GGM_{alk}+AGX**	**AGX**	**GGM_{alk}**	**acGX**
**DS_{ac}**	0.2	0	0	0	0.3
**$C2_{ac}$** (%)	70	n.d.	n.d.	n.d.	37
**$C3_{ac}$** (%)	30	n.d.	n.d.	n.d.	63
**M_w StC** (kDa)	37	45	44	38	31
**M_w LsC** (kDa)	39	20	17	23	26
**Monosaccharide composition** (mol%)					
**Fuc**	0.0	0.0	0.0	0.2	0.0
**Ara**	3.0	7.5	10.7	1.5	1.1
**Rha**	0.6	1.3	0.0	0.1	1.9
**Gal**	10.0	4.3	1.9	6.1	2.2
**Glc**	23.6	6.4	2.4	17.6	4.6
**Xyl**	0.7	50.6	70.6	1.9	74.8
**Man**	57.3	16.6	0.0	69.1	4.6
**mGlcA**	0.0	9.8	12.4	0.0	6.7
**GalA**	3.6	3.2	1.9	3.2	3.9
**GlcA**	1.4	0.4	0.3	0.3	0.3

Supplementary Table 2. Determination of the anisotropy ratios (*a_1*, *a_2*, and *a_3*). The anisotropy ratios were calculated at a CR≈0.1 from regular analysis by Method 1, as well as Method 2 where $F_n = 0$ was targeted to determine a_3.

**Sample**	**Method 1**			**Method 2**		
	**CR**	a_1	a_2	**CR**	a_1	a_3
BC	0.12	13	1.4	0.15	13	4
BC-acGGM	0.15	14	1.0	0.11	22	10
BC-AGX	0.13	7	0.7	0.13	22	10
BC-acGX	0.15	20	1.5	0.15	21	7

Supplementary Table 3. Values from uniaxial tensile testing on BC and *BC-acGGM* before and after a wash at pH 10 ("deac"). Different superscripts mean a significantly different value according to the ANOVA single factor analysis ($p<0.05$).

**Samples**	**Dry content (%)**		**ρ (mg/cm³)**		**E_{app} (MPa)**		**σ_{max} (MPa)**		**ε_{max} (%)**	
	**Avg.**	**±**	**Avg.**	**±**	**Avg.**	**±**	**Avg.**	**±**	**Avg.**	**±**
BC	8.4	*0.7*	82	*8*	2.4^{a}	*0.4*	1.4^{a}	*0.2*	53^{a}	*5*
deacBC	8.7	*0.2*	90	*5*	3.8^{b}	*0.6*	1.7^{b}	*0.1*	49^{a}	*1*
BC-acGGM	10.6	*1.0*	110	*6*	2.3^{a}	*0.3*	0.9^{c}	*0.1*	39^{b}	*2*
BC-deacGGM	9.5	*0.4*	100	*7*	2.6^{a}	*0.4*	0.9^{c}	*0.1*	35^{b}	*5*

Supplementary Table 4. Cellulose crystallinity and ratio of cellulose Iα to cellulose Iβ The values were determined by integration of corresponding peaks in the CP/MAS spectrum, standard deviation: ±2.

**Sample**	**Cryst. (%)**	**Iα (%)**	**Iβ (%)**
BC	79	47	53
BC-acGGM	83	47	53
BC-AGX	72	47	53
BC-acGGM+AGX	75	47	53
BC-GGM_{alk}*+AGX*	78	50	50
BC-acGX	81	53	47

Supplementary Table 5. Statistical analysis of mechanical properties. ANOVA single factor analysis for the mechanical properties presented in Table 1

Apparent Youngs Modulus, E_{app}:

Anova Single Factor for all samples in Table 1.

SUMMARY

Groups	*Count*	*Sum*	*Average*	*Variance*
BC	5	12.13	2.43	0.20
BC-acGGM	9	20.81	2.31	0.08
BC-AGX	8	2.96	0.37	0.01
BC-acGX	6	3.99	0.66	0.00
BC-acGGM+AGX	9	21.58	2.40	0.37
BC-GGMalk+AGX	8	5.36	0.67	0.03

ANOVA

Source of Variation	*SS*	*df*	*MS*	*F*	*P-value*	*F crit*
Between Groups	37.40	5	7.48	61.88	1.71E-17	2.46
Within Groups	4.71	39	0.12			
Total	42.11	44				

Superscript ”a” in Table 1:

SUMMARY

Groups	*Count*	*Sum*	*Average*	*Variance*
BC	5	12.13	2.43	0.20
BC-acGGM	9	20.81	2.31	0.08
BC-acGGM+AGX	9	21.58	2.40	0.37

ANOVA

Source of Variation	*SS*	*df*	*MS*	*F*	*P-value*	*F crit*
Between Groups	0.05	2	0.03	0.12	0.89	3.49
Within Groups	4.41	20	0.22			
Total	4.45	22				

Superscript ”c” in Table 1:

SUMMARY

Groups	*Count*	*Sum*	*Average*	*Variance*
BC-acGX	6	3.99	0.66	0.00
BC-GGMalk+AGX	8	5.36	0.67	0.03

ANOVA

Source of Variation	*SS*	*df*	*MS*	*F*	*P-value*	*F crit*
Between Groups	0.00	1.00	0.00	0.01	0.94	4.75
Within Groups	0.21	12.00	0.02			
Total	0.21	13.00				

Superscript ”b” and ”c” in Table 1:

SUMMARY

Groups	*Count*	*Sum*	*Average*	*Variance*
BC-AGX	8	2.96	0.37	0.01
BC-acGX	6	3.99	0.66	0.00
BC-GGMalk+AGX	8	5.36	0.67	0.03

ANOVA

Source of Variation	*SS*	*df*	*MS*	*F*	*P-value*	*F crit*
Between Groups	0.45	2.00	0.23	14.05	0.0002	3.52
Within Groups	0.31	19.00	0.02			
Total	0.76	21.00				

Stress at max, σ_{max}:

Anova Single Factor for all samples in Table 1.

SUMMARY

Groups	*Count*	*Sum*	*Average*	*Variance*
BC	5	7.16	1.43	0.03
BC-acGGM	9	8.32	0.92	0.02
BC-AGX	8	5.06	0.63	0.02
BC-acGX	6	5.17	0.86	0.01
BC-acGGM+AGX	9	8.48	0.94	0.04
BC-GGMalk+AGX	8	4.02	0.50	0.01

ANOVA

Source of Variation	*SS*	*df*	*MS*	*F*	*P-value*	*F crit*
Between Groups	3.17	5.00	0.63	28.18	5.67E-12	2.46
Within Groups	0.88	39.00	0.02			
Total	4.04	44.00				

Superscript ”c” in Table 1:

SUMMARY

Groups	*Count*	*Sum*	*Average*	*Variance*
BC-acGGM+AGX	9	8.48	0.94	0.04
BC-acGGM	9	8.32	0.92	0.02
BC-acGX	6	5.17	0.86	0.01

ANOVA

Source of Variation	*SS*	*df*	*MS*	*F*	*P-value*	*F crit*
Between Groups	0.02	2	0.01	0.44	0.65	3.47
Within Groups	0.59	21	0.03			
Total	0.61	23				

Superscript ”b” vs ”d” in Table 1:

SUMMARY

Groups	*Count*	*Sum*	*Average*	*Variance*
BC-AGX	8	5.06	0.63	0.02
BC-GGMalk+AGX	8	4.02	0.50	0.01

ANOVA

Source of Variation	*SS*	*df*	*MS*	*F*	*P-value*	*F crit*
Between Groups	0.07	1	0.07	5.32	0.04	4.60
Within Groups	0.18	14	0.01			
Total	0.25	15				

Superscript ”a” vs ”c” in Table 1:

SUMMARY

Groups	*Count*	*Sum*	*Average*	*Variance*
BC	5	7.16	1.43	0.03
BC-acGGM	9	8.32	0.92	0.02
BC-acGX	6	5.17	0.86	0.01
BC-acGGM+AGX	9	8.48	0.94	0.04

ANOVA

Source of Variation	*SS*	*df*	*MS*	*F*	*P-value*	*F crit*
Between Groups	1.13	3	0.38	13.47	1.98E-05	2.99
Within Groups	0.70	25	0.03			
Total	1.83	28				

Strain at max, ε_{max}:

Anova Single Factor for all samples in Table 1.

SUMMARY

Groups	*Count*	*Sum*	*Average*	*Variance*
BC	5	266.49	53.30	20.96
BC-acGGM	9	352.31	39.15	5.88
BC-AGX	8	695.28	86.91	86.38
BC-acGX	6	576.99	96.17	18.42
BC-acGGM+AGX	9	372.64	41.40	42.12
BC-GGMalk+AGX	8	570.59	71.32	106.21

ANOVA

Source of Variation	*SS*	*df*	*MS*	*F*	*P-value*	*F crit*
Between Groups	21516.54	5	4303.31	87.96	3.65E-20	2.46
Within Groups	1908.08	39	48.93			
Total	23424.63	44				

Superscript "b" in Table 1:

SUMMARY

Groups	*Count*	*Sum*	*Average*	*Variance*
BC-acGGM+AGX	9	372.64	41.40	42.12
BC-acGGM	9	352.31	39.15	5.88

ANOVA

Source of Variation	*SS*	*df*	*MS*	*F*	*P-value*	*F crit*
Between Groups	22.96	1	22.96	0.96	0.34	4.49
Within Groups	384.00	16	24.00			
Total	406.96	17				

Superscript ”a” vs “b” in Table 1:

SUMMARY

Groups	*Count*	*Sum*	*Average*	*Variance*
BC-acGGM+AGX	9	372.64	41.40	42.12
BC-acGGM	9	352.31	39.15	5.88
BC	5	266.49	53.30	20.96

ANOVA

Source of Variation	*SS*	*df*	*MS*	*F*	*P-value*	*F crit*
Between Groups	686.61	2	343.30	14.68	0.00012	3.49
Within Groups	467.85	20	23.39			
Total	1154.46	22				

Superscript ”c” vs “e” in Table 1:

SUMMARY

Groups	*Count*	*Sum*	*Average*	*Variance*
BC-AGX	8	695.28	86.91	86.38
BC-acGX	6	576.99	96.17	18.42

ANOVA

Source of Variation	*SS*	*df*	*MS*	*F*	*P-value*	*F crit*
Between Groups	293.67	1	293.67	5.06	0.044	4.75
Within Groups	696.76	12	58.06			
Total	990.44	13				

Superscript ”c” vs “d” in Table 1:

SUMMARY

Groups	*Count*	*Sum*	*Average*	*Variance*
BC-AGX	8	695.28	86.91	86.38
BC-GGMalk+AGX	8	570.59	71.32	106.21

ANOVA

Source of Variation	*SS*	*df*	*MS*	*F*	*P-value*	*F crit*
Between Groups	971.72	1	971.72	10.09	0.007	4.60
Within Groups	1348.11	14	96.29			
Total	2319.84	15				

Supplementary References

1 Baskin, T. I. ANISOTROPIC EXPANSION OF THE PLANT CELL WALL. *Annual Review of Cell and Developmental Biology* **21**, 203-222, doi:10.1146/annurev.cellbio.20.082503.103053 (2005).

2 Lopez-Sanchez, P. *et al.* Micromechanics and Poroelasticity of Hydrated Cellulose Networks. *Biomacromolecules* **15**, 2274-2284, doi:10.1021/bm500405h (2014).

3 Mow, V. C., Kuei, S. C., Lai, W. M. & Armstrong, C. G. Biphasic Creep and Stress Relaxation of Articular Cartilage in Compression: Theory and Experiments. *Journal of Biomechanical Engineering* **102**, 73-84, doi:10.1115/1.3138202 (1980).

4 Cohen, B., Lai, W. M. & Mow, V. C. A Transversely Isotropic Biphasic Model for Unconfined Compression of Growth Plate and Chondroepiphysis. *Journal of Biomechanical Engineering* **120**, 491-496, doi:10.1115/1.2798019 (1998).

5 Bonilla, M. R., Lopez-Sanchez, P., Gidley, M. J. & Stokes, J. R. Micromechanical model of biphasic biomaterials with internal adhesion: Application to nanocellulose hydrogel composites. *Acta Biomaterialia* **29**, 149-160, doi:https://doi.org/10.1016/j.actbio.2015.10.032 (2016).

6 Bursać, P. M., Obitz, T. W., Eisenberg, S. R. & Stamenović, D. Confined and unconfined stress relaxation of cartilage: appropriateness of a transversely isotropic analysis. *Journal of Biomechanics* **32**, 1125-1130, doi:https://doi.org/10.1016/S0021-9290(99)00105-0 (1999).
